# Parenthood and risk of hip fracture: a 10-year follow-up prospective study of middle-aged women and men in China

**DOI:** 10.1007/s00198-019-05185-2

**Published:** 2019-11-25

**Authors:** K. Peng, P. Yao, L. Yang, C. Kartsonaki, D. Bennett, M. Tian, Y. Guo, Z. Bian, Y. Chen, Z. Chen, M. Woodward, R. Ivers, R. Clarke

**Affiliations:** 1 School of Public Health, The University of Sydney, Sydney, Australia; 2 The George Institute for Global Health, UNSW, Sydney, Australia; 3 Clinical Trial Service Unit and Epidemiological Studies Unit, Big Data Institute, University of Oxford, Old Road Campus, Oxford, OX3 7LF UK; 4 The George Institute for Global Health at Peking University Health Science Center, Beijing, China; 5 Chinese Academy of Medical Sciences, Beijing, China; 6 The George Institute for Global Health, Oxford University, Oxford, UK; 7 Department of Epidemiology, Johns Hopkins University, Baltimore, MD USA; 8 School of Public Health and Community Medicine, UNSW, Sydney, Australia

**Keywords:** Hip fracture, Number of children, Parenthood, Sex

## Abstract

**Summary:**

This prospective study of Chinese adults demonstrated an inverse J-shaped association of number of children with risk of hip fracture in both men and postmenopausal women aged 50 years or older. Women with 2 or 3 children and men with 4 children had the lowest risk of hip fracture.

**Introduction:**

Women have higher absolute risks of fracture than men, which is believed to reflect differences in oestrogen exposure. The aim of this study was to compare the associations of number of children with risk of hip fracture between men and women aged over 50 years.

**Methods:**

The China Kadoorie Biobank (CKB) recruited 133,399 women and 110,296 men, aged 50 years or older between 2004 and 2008. During 10-year follow-up, 2068 participants (1394 women and 674 men) suffered a hip fracture. Cox regression analysis was used to estimate sex-specific adjusted hazard ratios (HRs) and 95% CI for incident hip fracture.

**Results:**

Over 98% of both subsets of men and women aged 50 or older reported having children. Women who had 2 or 3 children had the lowest risks of hip fracture compared with other groups. Compared with nulliparous women, the adjusted HR for hip fracture were 0.89 (95% CI; 0.72, 1.10) for 1 child, 0.79 (0.70, 0.90) for 2 children, 0.79 (0.72, 0.87) for 3 children, 0.81 (0.72, 0.91) for 4 children, and 0.95 (0.83, 1.10) for those with 5 or more children. The associations of number of children with hip fracture were broadly consistent in men of a similar age.

**Conclusions:**

The concordant effects of the number of children with risk of hip fracture between men and women suggest that the lower risks in multiparous women are not due to differences in oestrogen exposure or other biological effects, but may reflect residual confounding by socioeconomic or lifestyle factors.

## Introduction

Hip fracture is a major public health problem associated with a high morbidity and mortality in both developed and developing countries. It has been estimated that about 50% of all hip fractures will occur in Asian populations by 2050 [1–4]. Women start to suffer bone loss between 30 and 39 years, and their risks of fracture increase substantially after the menopause. Worldwide, 1 in 3 women and 1 in 5 men over age 50 years will experience an osteoporotic fracture with hip fractures being the most serious type of fracture [5–7].

The number of biological children has been linked with higher risks of hip fracture [8–12]. Pregnancy and hormonal changes in women are believed to have adverse effects on bone health in women. Maternal skeletal calcium is extracted to facilitate foetal and neo-natal growth [13–15]. However, bone loss during pregnancy and breastfeeding is largely restored after weaning [16, 17], but whether this is also true for women with a large family, or whether there are any long-term effects of parity on bone health is not fully understood.

The results of previous studies of the association of parity with fracture among postmenopausal women have been conflicting [8–12, 18, 19]. Two studies conducted in USA and one in Finland reported that higher levels of parity were associated with lower risks of hip fracture in later life in women [10–12]. However, studies from Norway and Denmark have reported no association of parity with hip fracture [18, 19]. In contrast, other studies conducted in Japan and Denmark reported that there was an inverse J-shaped association of parity with risk of hip fracture [9, 20]. Little is known about the associations of parity with risk of hip fracture in postmenopausal women living in China.

We examined the associations of number of children with risk of incident hip fracture in both older women and men in the China Kadoorie Biobank (CKB). We compared the sex differences in the associations of hip fracture with number of children to assess whether any differences in such associations might be explained by differences in oestrogen exposure between men and women.

## Methods

### Baseline survey

Details of the study design and procedures used in CKB have been previously published [21]. Briefly, 512,891 adults (210,222 men and 302,669 women) aged 30–79 years were recruited from 10 areas (5 urban and 5 rural) in China between 2004 and 2008. Data collected are as follows: general demographic, socioeconomic, dietary and other lifestyle factors (e.g. smoking, tea, alcohol drinking, and physical activity), passive smoking and indoor air pollution, personal and family medical history, sleeping and psychological wellbeing. Women’s reproductive factors (e.g. age at menarche, age at menopause (in postmenopausal women), number of children (restricted to biological children), breastfeeding history, hysterectomy/ovariectomy history, use of oral contraceptives) were collected in women by trained health workers using a laptop-based questionnaire. Ethics approval was obtained from relevant international, national and local authorities, and all participants provided written informed consent.

### Follow-up for morbidity and mortality

Study participants were followed up for cause-specific morbidity and mortality by linkage with regional disease and death registers and with the national health insurance system. Active follow-up was also performed on an annual basis to minimize any loss to follow-up. All deaths and diseases were coded using the International Classification of Diseases (ICD-10) and the coding was blinded to baseline exposures. The primary endpoint was incident hip fracture as defined by ICD-10 codes S72.0–S72.9.

The present study involved postmenopausal women, aged 50 years or over (*n* = 145,172), men aged 50 years or over (*n* = 121,428), with complete data on the number of children at baseline. Participants with a self-reported history of cancer were excluded (*n* = 1984 [58.1% women]), as were 20,980 individuals with a self-reported history of fracture, leaving a total of 110,296 men and 133,399 women for the present analyses.

### Statistical analysis

Participant characteristics at study baseline are presented as means (SD) for continuous variables and as percentages for categorical variables. Cox proportional hazards models with time in study as the timescale were used to estimate sex-specific hazard ratios (HRs) and 95% CIs for the association of number of children with risk of fatal or non-fatal hip fracture. Multivariable Cox models were stratified by region and age-at-risk (model 1), and were additionally adjusted for education (≤ primary, ≥ secondary), household income (< 5000, 5000–19,999, and ≥ 20,000 yuan) and marital status (model 2), and additionally for history of diabetes, chronic obstructive pulmonary disease (COPD), stroke, coronary heart disease (CHD), body mass index (BMI), self-rated health, smoking status, alcohol consumption, dairy intake, fruit intake, soybean products intake, calcium or zinc or iron intake, and physical activity (MET-h/day) (model 3). A further analysis was performed in women to adjust for reproductive factors including age at first birth, age at menopause and duration of breastfeeding (model 4). Group-specific variances were used for variables with 3 or more categories to facilitate comparisons between any two categories [22].

Pre-specified subgroup analyses of the effects of parenthood on risk of fracture were conducted by region, age and BMI in men and women, respectively. Additional sensitivity analyses included the following: (i) excluding individuals who had more than ten children (to exclude extreme effects); (ii) analysis of number of live births (to exclude neonatal deaths) with risk of hip fracture in women; and (iii) exclusion of individuals who had reported having a hysterectomy or oophorectomy at baseline (to exclude artefacts of differences in oestrogen levels due to surgery). Analyses were performed using SAS version 9.3 (SAS institute, Cary, NC, USA) and R version 3.5.1 (R Foundation for Statistical Computing, Vienna, Austria)

## Results

Among the 243,695 participants included, the mean (SD) age at baseline was 60 (6.9) years and 55% were women. In this subset of CKB participants, 98.8% of women and 97.6% of men reported having any children, and one-third of both women (Table 1) and men had at least two children (Table 2). In both sexes, individuals who had one child were generally younger, better educated and more likely to live in urban areas compared with those who had no children or had more than one child. Physical activity levels were generally lower among men who had more children. Men reported higher proportions of smoking and alcohol consumption compared with women.

**Table 1:** Baseline characteristics of women by number of children

	Total	Number of children					
		0	1	2	3	4	≥ 5
*N* (%)	133,399	1561 (1.2)	19,780 (14.8)	43,193 (32.4)	36,333 (27.2)	19,605 (14.7)	12,927 (9.7)
Hip fractures (per 1000)	1394 (10.4)	23 (14.7)	100 (5.1)	318 (7.4)	416 (11.5)	296 (15.1)	241 (18.6)
Rural, %	53.6	50.4	28.5	52.0	59.8	61.8	67.8
Age, years	60.3 (6.7)	61.6 (7.3)	54.9 (4.6)	57.7 (5.1)	61.3 (6.1)	64.5 (6.2)	67.5 (5.8)
Education primary or lower, %	74.0	66.2	42.4	69.6	81.6	88.9	94.5
Married, %	82.1	70.3	89.1	88.2	81.7	74.6	65.6
Low household income, %	14.7	18.1	6.2	8.6	15.6	23.7	31.5
Current smoking, %	5.9	7.3	5.6	4.6	6.1	7.5	7.8
Weekly alcohol use, %	2.3	2.4	2.8	2.2	2.2	2.2	2.4
Weekly dairy consumption, %	67.2	63.0	52.0	68.0	71.0	71.2	72.1
Weekly fruit consumption, %	6.8	6.9	3.4	5.4	7.1	9.2	12.5
Weekly soy consumption, %	58.2	58.5	66.3	62.9	57.2	50.8	43.9
Regular calcium consumption, %	10.7	9.7	13.3	10.3	10.4	10.2	10.1
Medical history, %							
Diabetes	9.8	8.1	7.9	9.2	10.1	11.1	11.4
COPD	8.7	10.9	6.6	7.2	8.9	11.3	12.4
CHD	5.7	6.6	4.5	5.1	6.2	6.5	6.7
Stroke	2.4	2.6	1.6	2.0	2.5	3.1	3.2
Poor self-rated health, %	62.1	68.0	59.1	59.0	62.4	66.4	69.3
Physical activity, MET h/day	12.9 (8.4,20.8)	11.2 (8.4,17.7)	14.0 (8.9,21.8)	14.0 (9.7,23.0)	13.1 (8.9,21.0)	11.3 (8.4,17.8)	10.4 (8.4,15.1)
BMI, kg/m^2^	24.0 (3.7)	23.5 (3.8)	24.3 (3.5)	24.1 (3.6)	23.9 (3.7)	23.8 (3.8)	23.5 (3.8)

Values are percentages for categorical variables, and means (SD) or median (25th and 75th percentiles) for continuous variables

*BMI*, body mass index; *MET*, metabolic equivalent of task

**Table 2:** Baseline characteristics of men by number of children

	Total	Number of children					
		0	1	2	3	4	≥5
*N* (%)	110,296	2657 (2.4)	23,211 (21.0)	36,790 (33.4)	27,160 (24.6)	12,964 (11.8)	7514 (6.8)
Hip fracture (per 1000)	674 (6.1)	26 (9.8)	85 (3.7)	200 (5.4)	188 (6.9)	101 (7.8)	74 (9.6)
Rural, %	57.3	81.5	32.5	58.4	64.8	67.5	74.9
Age, years	60.6 (7.2)	61.3 (7.2)	55.5 (5.1)	59.0 (6.0)	62.6 (6.7)	65.6 (6.5)	68.2 (5.9)
Education primary or lower, %	57.2	82.5	38.6	56.9	61.2	67.4	75.4
Married, %	91.1	33.7	93.4	94.4	92.6	89.7	85.4
Low household income, %	12.7	45.7	6.1	7.7	13.7	21.1	27.8
Current smoking, %	67.2	70.2	69.5	67.9	66.2	64.2	65.1
Weekly alcohol use, %	30.4	23.6	40.6	31.8	26.8	23.3	19.8
Weekly dairy consumption, %	68.9	84.2	60.5	71.6	70.4	69.7	69.6
Weekly fruit consumption, %	8.9	18.6	7.8	7.9	8.7	9.8	12.6
Weekly soy consumption, %	62.4	51.5	69.8	65.8	59.9	55.3	48.2
Regular calcium consumption, %	6.5	4.2	5.3	6.1	7.2	7.9	8.3
Medical history, %							
Diabetes	7.3	5.2	8.0	7.2	7.3	7.5	6.3
COPD	12.6	19.0	8.9	11.2	13.9	15.8	18.3
CHD	4.3	3.1	3.1	3.9	4.9	6.0	5.9
Stroke	3.7	2.8	2.4	3.4	4.4	5.0	5.2
Poor self-rated health, %	54.5	66.1	51.2	53.0	55.3	57.7	60.1
Physical activity, MET h/day	14.4 (6.9, 27.2)	14.3 (8.4, 25.7)	18.2 (10.1, 30.8)	15.8 (7.6, 30.1)	12.6 (6.1, 24.7)	10.6 (5.6, 21.1)	8.9 (4.5, 18.4)
BMI, kg/m^2^	23.2 (3.2)	21.8 (3.1)	23.9 (3.2)	23.3 (3.2)	23.0 (3.2)	22.8 (3.3)	22.5 (3.3)

Values are means (SD) or median (25th and 75th percentiles) for continuous variables and percentages for categorical variables

*MET*, metabolic equivalent of task

**Fig. 1:**
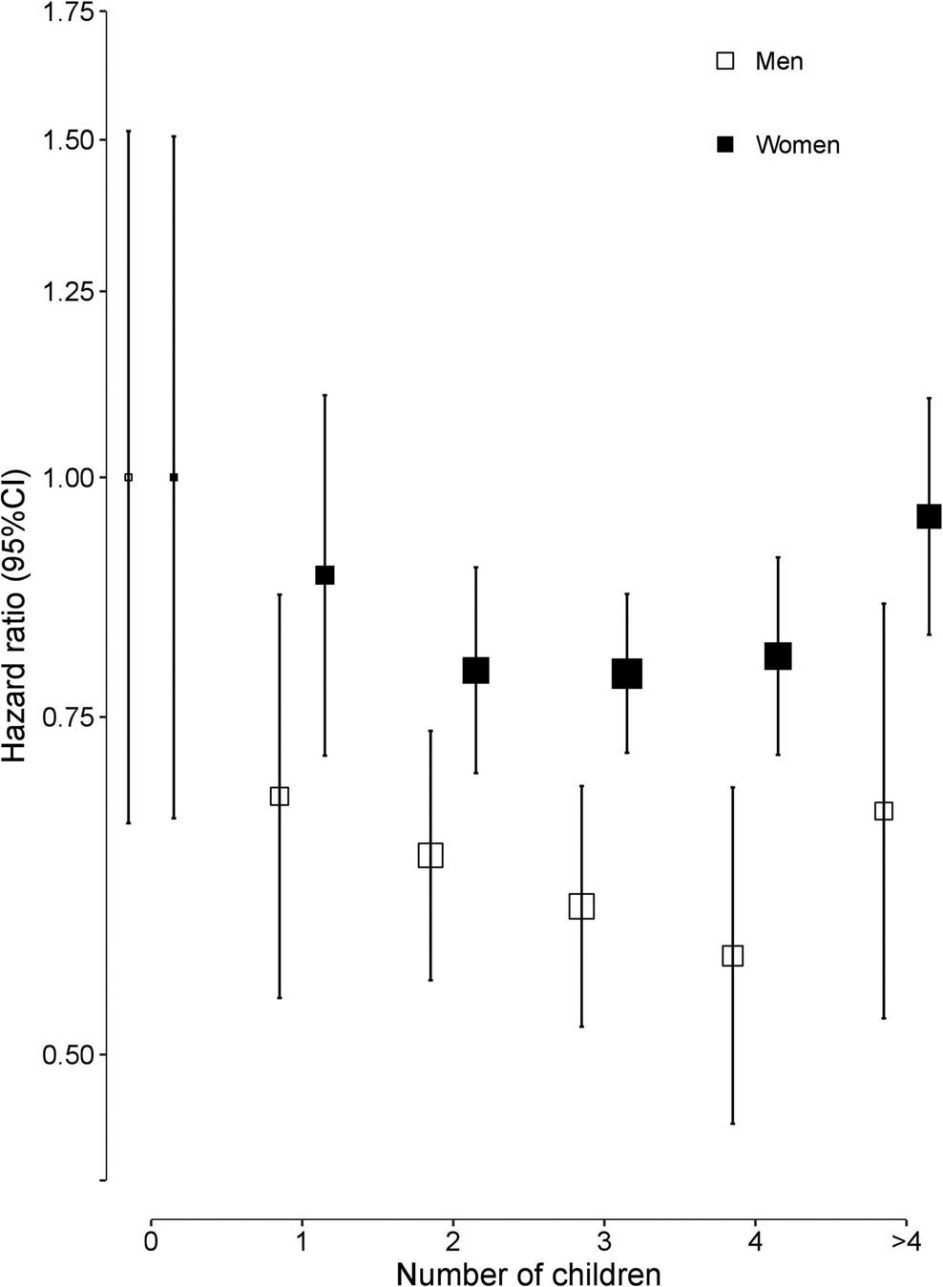
Adjusted hazard ratios (HRs) for risk of incident hip fracture associated with number of children in women and men. Data for women (black squares) include 1394 fracture cases and for men (white squares) include 674 fracture cases. Models were stratified by age at risk and study areas, and additionally adjusted for level of attained education, household income, marital status, diabetes, COPD, stroke, CHD, self-rated health, smoking status, alcohol use, dairy intake, fruit intake, soy intake, calcium intake, physical activity and body mass index (BMI). Each square has an area inversely proportional to the standard error of the log HR. Vertical lines indicate the corresponding 95% CI

**Fig. 2:**
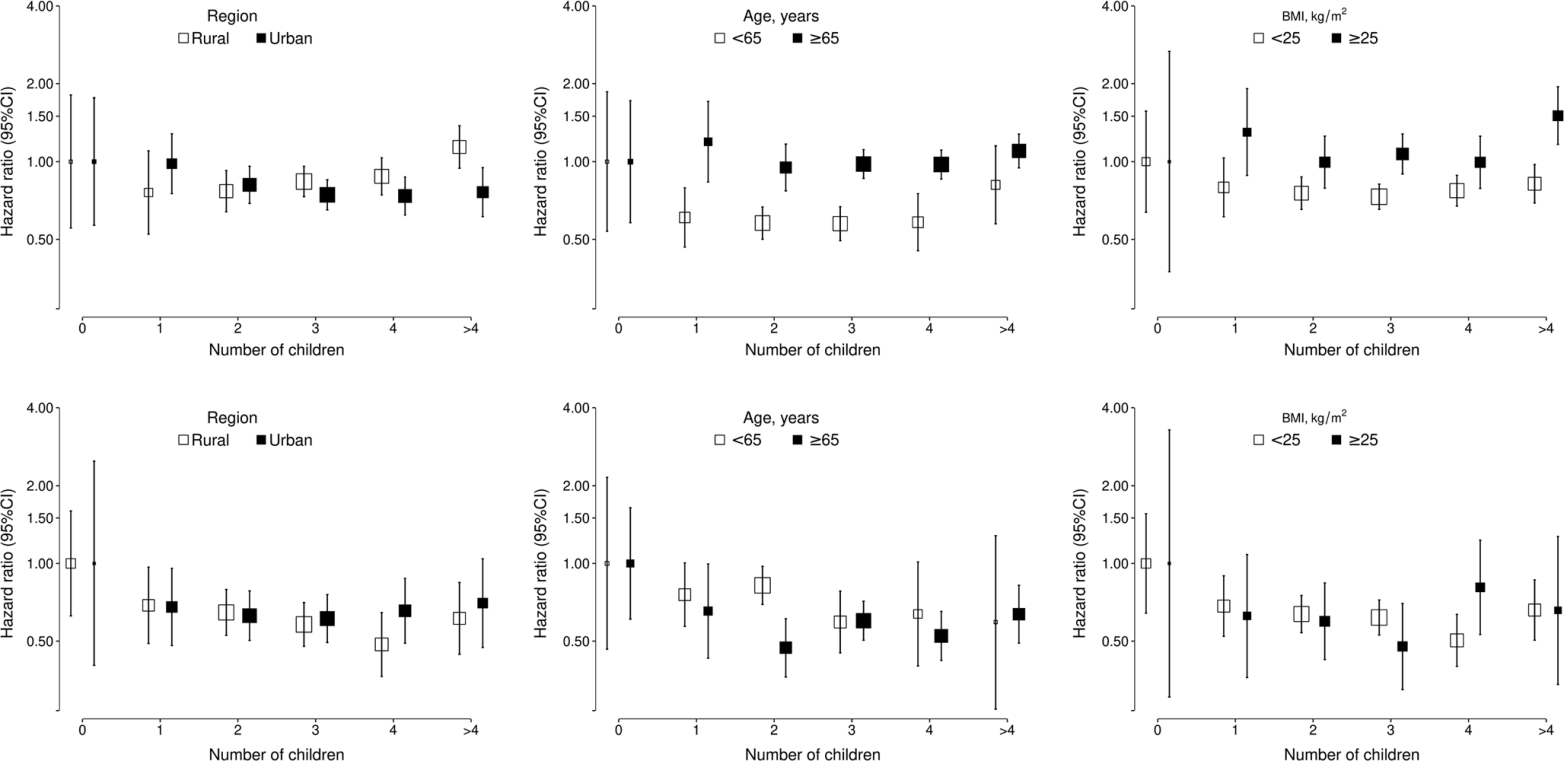
Adjusted hazard ratios (HRs) and 95% CIs for risk of incident hip fracture associated with number of children in women (top panels) and men (bottom panels) in population subgroups. All models were stratified by age at risk and study area, and adjusted for level of attained education, household income, marital status, diabetes, COPD, stroke, CHD, self-rated health, smoking status, alcohol use, dairy intake, fruit intake, soy intake, calcium intake, physical activity and body mass index

**Fig. 3:**
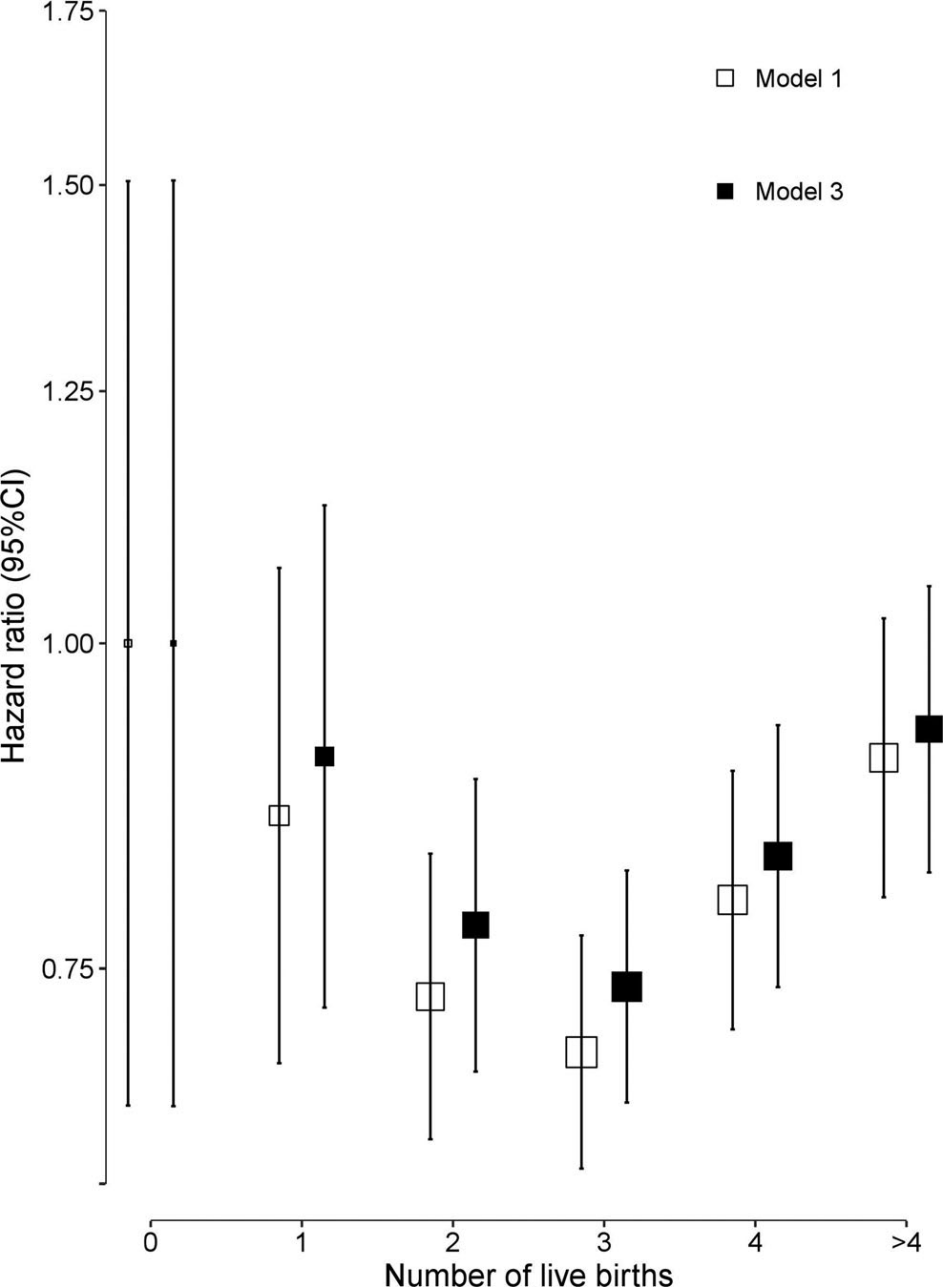
Adjusted hazard ratios (HRs) for risk of incident hip fracture associated with number of live births in women. Models were stratified by age at risk and region (model 1, white squares), and additionally adjusted for level of attained education, household income, marital status, diabetes, COPD, stroke, CHD, self-rated health, smoking status, alcohol use, dairy intake, fruit intake, soy intake, calcium intake, physical activity and body mass index (model 3, black squares)

During a median of 10.0 years (interquartile range 9.0–11.0) of follow-up, 2068 (1394 women and 674 men) incident cases of hip fracture were recorded. Overall, nulliparous women tended to have a higher risk of hip fracture (adjusted HR 1.25 [95% CI 0.83, 1.89]) compared with parous women in the age- and region-stratified models, which were attenuated slightly (HR 1.20, (95% CI 0.80, 1.82)) after further adjustment for socioeconomic and lifestyle factors (Table 3). An inverse J-shaped association was found between the number of children and risk of hip fracture among women (Fig. 1). Compared with nulliparous women, the adjusted HRs (95% CI) were 0.86 (0.69, 1.06) for women with 1 child, 0.75 (0.67, 0.85) for 2 children, 0.75 (0.69, 0.83) for 3 children, 0.79 (0.70, 0.88) for 4 children and 0.94 (0.81, 1.08) for 5 or more children. Adjustment for socioeconomic status, medical history and lifestyle behaviours only slightly attenuated these associations (Table 3). The associations of the number of children with fatal or non-fatal hip fractures were broadly consistent between different population subgroups defined by region, age, BMI, and age at first birth, and in sensitivity analyses among the subset of women after excluding those who had more than 10 children, or restricting analyses to the number of live births or those with no history of hysterectomy or oophorectomy (Figs. 2 and 3). However, additional adjustment for age at first birth, age at menopause and duration of breastfeeding completely attenuated the associations of number of children with risk of fracture (Table 3).

**Table 3:** HR (95% CI) for risk of incident hip fracture associated with number of children in women and in men before and after adjustment for confounding factors

	Childless vs having children	Number of children					
		No children	1 child	2 children	3 children	4 children	≥ 5 children
Women							
Rate of hip fracture		23/1561	100/19,780	318/43,193	416/36,333	296/19,605	241/12,927
Model I	1.25 (0.83,1.89)	1.00 (0.66,1.51)	0.86 (0.69, 1.06)	0.75 (0.67,0.85)	0.75 (0.69,0.83)	0.79 (0.70,0.88)	0.94 (0.81,1.08)
Model II	1.24 (0.82,1.87)	1.00 (0.66,1.51)	0.86 (0.69, 1.07)	0.76 (0.67,0.86)	0.76 (0.69,0.84)	0.80 (0.71,0.90)	0.94 (0.82,1.09)
Model III	1.20 (0.80,1.82)	1.00 (0.66,1.51)	0.89 (0.72, 1.10)	0.79 (0.70,0.90)	0.79 (0.72,0.87)	0.81 (0.72,0.91)	0.95 (0.83,1.10)
Model IV	0.92 (0.55, 1.56)	1.00 (0.59, 1.69)	1.05 (0.84, 1.32)	1.02 (0.91, 1.16)	1.05 (0.95, 1.15)	1.07 (0.95, 1.21)	1.28 (1.11, 1.48)
Men							
Rate of hip fracture		26/2657	85/23,211	200/36,790	188/27,160	101/12,964	74/7514
Model I	1.72 (1.16,2.56)	1.00 (0.68,1.47)	0.62 (0.49, 0.79)	0.58 (0.50,0.67)	0.56 (0.49,0.65)	0.54 (0.44,0.66)	0.65 (0.51,0.83)
Model II	1.69 (1.11,2.57)	1.00 (0.66,1.51)	0.65 (0.51, 0.82)	0.60 (0.52,0.70)	0.57 (0.50,0.66)	0.54 (0.44,0.66)	0.65 (0.51,0.83)
Model III	1.61 (1.06,2.46)	1.00 (0.66,1.52)	0.68 (0.54, 0.87)	0.64 (0.55,0.74)	0.60 (0.52,0.69)	0.56 (0.46,0.69)	0.67 (0.52,0.86)

Model I, stratified by age at risk and study area; Model II, model I + adjusted for education, household income and marital status; Model III, model II + adjusted for diabetes, COPD, stroke, CHD, self-rated health, smoking status, alcohol use, dairy intake, fruit intake, soy intake, calcium intake, physical activity and body mass index; Model IV, Model III + age at first birth, age at menopause and duration of breast feeding

The associations of number of children with risk of hip fracture were similar in both men and in women. Childless men were more likely to sustain a hip fracture in later life compared with men who had at least one child (1.72, 95% CI; 1.16, 2.56) in analyses stratified by age and region, and after further adjustment for socioeconomic factors, medical history and lifestyle behaviours (1.61 [95% CI; 1.06, 2.46]; Table 3). Compared with men with no children, the adjusted hazard ratios (95% CI) for men with 1 child was 0.62 (0.49, 0.79), 0.58 (0.50, 0.67) for 2 children, 0.56 (0.49, 0.65) for 3 children, 0.54 (0.44, 0.66) for 4 children and 0.65 (0.51, 0.83) for 5 or more children. Consistent with the results in women, the associations were only slightly attenuated after additional adjustment for socioeconomic factors, medical history and lifestyle behaviours (Table 3). No substantial differences were observed between groups after incremental adjustment for a wide range of confounding factors (Table 3). Likewise, the associations were also similar when the analyses were restricted to men with up to 10 children.

The fully adjusted HR of hip fracture comparing those without children with those with at least one child was significant in men ([HR 1.61, 95% CI; 1.06, 2.46] but not in women [HR 1.20, 95% CI; 0.80, 1.82]) in comparable analyses, albeit there were no significant heterogeneity by sex (*p* for heterogeneity = 0.3). The associations of number of children with risk of fracture in comparable analyses were broadly consistent between men and women.

## Discussion

This report of a prospective study of 243,695 men and postmenopausal women aged 50 years or older who were recruited from 10 regions of China demonstrated an inverse J-shaped association between number of children and risk of hip fracture in both women and men. The lowest risks of hip fracture were observed among men with 4 children and women with 2 or 3 children. The concordant shape and strength of associations between women and men suggest that association of parenthood with hip fracture is not due to any biological effects, but may reflect confounding by socioeconomic or lifestyle factors. However, because of the limitations of available measures on socioeconomic status; it was not possible to fully exclude the effects of residual confounding by socioeconomic or lifestyle factors.

Previous studies have reported conflicting results for the associations of number of children with risk of hip fracture. The reasons for the discrepant results of different studies may reflect the relative smaller sample sizes and the inconsistent methods used to record the number of children, and the heterogeneity in the age ranges of participants. In two prospective studies, nulliparous women were also found to be more likely to suffer a hip fracture later in life [12, 22], but other studies have reported no significant differences in the risk of hip fracture between nulliparous women and parous women who had children [18, 23]. A meta-analysis of 19 reports, involving 217,295 women and 26,525 fracture cases, reported that increasing number of parity was inversely associated with risk of fracture [8]. In the present study, the risk of hip fracture was higher among nulliparous women, albeit differences were less pronounced than in previous studies.

An inverse J-shaped association between number of children and hip fracture was also reported in studies conducted in Japan and Denmark, consistent with the findings of the present study. The Japanese study recruited 2987 women aged 32 years or greater (mean age 58.6 years), but had only 49 cases with fracture during the 14-year follow-up period [20]. However, unlike the present study, some of the participants in the Japanese study had not reached the menopause. Moreover, a Danish study of 1708 women aged 66 to 99 years also reported an inverse J-shaped association between number of children and risk of hip fracture after adjustment for age and BMI. However, no statistically significant differences were observed with risk of hip fracture between women with a different number of children [9]. In contrast, an inverse association between number of children and risk of hip fracture was observed among women in three studies conducted in USA and Finland [10–12]. Two American studies reported an inverse association between the number of children and risk of hip fracture in women. One study reported that women with 3 or more children had the lowest risk of hip fracture in the age-stratified model [10], and another study reported that nulliparous women had a 44% higher risk of hip fracture (hazard ratio, 1.44; 95% CI, 1.17–1.78) [12]. Moreover, a Finnish study of 1959 women reported that those who had 3 or more children had a 50% (95% CI; 32–79) lower risk of hip fracture compared with nulliparous women [11]. Likewise, a meta-analysis of 19 studies, involving 217,295 women and 26,525 fracture cases, reported that increasing number of children was linearly associated with a reduced risk of hip fracture. Further adjustment for age at first birth, age at menopause and duration of breastfeeding completely attenuated the association of number of children with risk of fracture.

Higher levels of oestrogen are believed to be protective for women in helping to prevent bone loss and hip fractures [24–26]. However, there is little evidence of any direct protective effect from changes in oestrogen levels during pregnancy on subsequent risk of hip fracture in later life. It has been postulated that the bone structure of the hip changes after delivery, reducing the subsequent risk of hip fracture [27, 28]. Other studies have reported that bone loss takes place during pregnancy because the calcium needed for growth of foetal skeleton is provided by the maternal skeleton [13–15].

The present study demonstrated an inverse J-shaped association between the number of children and risk of hip fracture in men aged 50 years and over. However, there were no statistical differences observed between men and women for the associations between number of children and risk of hip fracture. An inverse J-shaped relationship between number of children and risk of hip fracture was also demonstrated in the Danish twin study with no difference between both sexes in older Danish people aged 66 and over [9]. The concordant results observed between men and women suggest that rather than being due to the biological mechanisms of childbearing, socioeconomic, cultural and psychological factors correlated with family size may explain the associations of number of children with risk of hip fracture. Dietary behaviour could also explain some of these associations, but the proportion of fresh fruit consumers and intake of calcium was higher in both women and men with a larger family size in this study population. However, larger family size may be correlated with stress to the parents which could increase their risk of falls which was the main cause of hip fracture [29]. The large sex differences in the incidence of hip fracture may reflect the different prevalence of osteoporosis and falls between women and men [30, 31].

### Strengths and limitations

The chief strengths of the present study include the large sample size, comprehensive information on reproductive factors, socio-demographic, medical history and lifestyle factors of participants. The participants were recruited from 10 different areas (5 urban and 5 rural) from north to south in China. The analyses in the study adjusted for several confounders, including socio-demographic, physical and lifestyle characteristics, and medical history. Limitations of the present study included combined analysis of all hip fractures (i.e. both fragility and non-fragility hip fractures); absence of information on bone mineral density [12], or consumption and status of vitamin D, which are widely believed to be associated with risk of hip fracture); relatively small sample size of nulliparous participants; and lack of information on reasons why the participants may have been nulliparous. Childlessness may not only be associated with socio-economic factors, but may also be an indicator of poor health. A further limitation of this study was the inability to compare the associations of number of children with risk of fracture in pairs of men and women from the same family when addressing the associations of number of children with risk of fracture to address residual confounding by social class.

## Conclusions

The present study demonstrated that there was an inverse J-shaped association of the number of children with risk of hip fracture among postmenopausal women and men aged 50 years or older. Women and men with no children had a higher risk of hip fracture compared with those who had at least one child. Women with 2 or 3 children and men with 4 children had the lowest risk of hip fracture in each sex, respectively. The concordant findings of associations of number of children with risk of hip fracture in men and women suggest that the risks are not due to any biological factors, but may instead reflect differences in socioeconomic, cultural or physical factors relevant to the number of children. Hence, the results of the present study highlight the need for further studies of socioeconomic, cultural or physical factors as biological explanations of the relation between family size and risk of hip fracture in men and women. In addition, further analyses of the associations of additional reproductive factors with risk of hip fracture among postmenopausal women are needed to understand sex differences in the risk of fractures in high-risk populations.

## Data Availability

RCl, DB, LL and ZC had full access to all the data in the study and take responsibility for the integrity of all data and accuracy of the data analysis. Data from the baseline survey, first resurvey, and cause-specific mortality are available to all bona fide researchers (www.ckbiobank.org). Additional data are also made available on a collaborative basis by contacting the study investigators. All data requests are reviewed monthly by the CKB Data Access Committee, which is composed of senior scientists from Beijing and Oxford.

## References

[1] Cooper C, Campion G, Melton LR (1992). Hip fractures in the elderly: a world-wide projection. Osteoporos Int.

[2] Broderick J et al (2013) Osteoporotic hip fractures: the burden of fixation failure. Sci World J 2013 10.1155/2013/515197 PMC3580900 23476139

[3] Yang Y (2015). Inpatient cost of treating osteoporotic fractures in mainland China: a descriptive analysis. ClinicoEcon Outcomes Res.

[4] Qu B (2014). The economic burden of fracture patients with osteoporosis in western China. Osteoporos Int.

[5] Melton LJ (1998). Bone density and fracture risk in men. J Bone Miner Res.

[6] Melton LJ (1992). Perspective how many women have osteoporosis?. J Bone Miner Res.

[7] Kanis J (2000). Long-term risk of osteoporotic fracture in Malmö. Osteoporos Int.

[8] Wang Q (2016). Parity and osteoporotic fracture risk in postmenopausal women: a dose-response meta-analysis of prospective studies. Osteoporos Int.

[9] Petersen HC (2002). Reproduction life history and hip fractures. Ann Epidemiol.

[10] Paganini-Hill A (2005). Menstrual and reproductive factors and fracture risk: the Leisure World Cohort Study. J Women's Health.

[11] Kauppi M (2011). Parity and risk of hip fracture in postmenopausal women. Osteoporos Int.

[12] Hillier TA (2003). Nulliparity and fracture risk in older women: the study of osteoporotic fractures. J Bone Miner Res.

[13] Black A (2000). A detailed assessment of alterations in bone turnover, calcium homeostasis, and bone density in normal pregnancy. J Bone Miner Res.

[14] Kovacs CS (2011). Calcium and bone metabolism disorders during pregnancy and lactation. Endocrinol Metab Clin.

[15] Prentice A (2000). Calcium in pregnancy and lactation. Annu Rev Nutr.

[16] Karlsson MK, Ahlborg HG, Karlsson C (2005). Maternity and bone mineral density. Acta Orthop.

[17] Pearson D (2004). Recovery of pregnancy mediated bone loss during lactation. Bone.

[18] Hundrup YA (2005). Risk factors for hip fracture and a possible effect modification by hormone replacement therapy. The Danish Nurse Cohort Study. Eur J Epidemiol.

[19] Bjørnerem Å (2011). Breastfeeding protects against hip fracture in postmenopausal women: the Tromsø study. J Bone Miner Res.

[20] Fujiwara S (1997). Risk factors for hip fracture in a Japanese cohort. J Bone Miner Res.

[21] Chen Z (2011). China Kadoorie Biobank of 0.5 million people: survey methods, baseline characteristics and long-term follow-up. Int J Epidemiol.

[22] Taylor BC (2004). Long-term prediction of incident hip fracture risk in elderly white women: study of osteoporotic fractures. J Am Geriatr Soc.

[23] Crandall CJ (2017). Associations of parity, breastfeeding, and fractures in the Women’s Health Observational Study. Obstet Gynecol.

[24] Cummings SR (1998). Endogenous hormones and the risk of hip and vertebral fractures among older women. N Engl J Med.

[25] Sipilä S (2006). Endogenous hormones, muscle strength, and risk of fall-related fractures in older women. J Gerontol Ser A Biol Med Sci.

[26] McEwen BS (1999). The molecular and neuroanatomical basis for estrogen effects in the central nervous system. J Clin Endocrinol Metab.

[27] Oliveri B (2004). Mineral and bone mass changes during pregnancy and lactation. Nutrition.

[28] Specker B, Binkley T (2005). High parity is associated with increased bone size and strength. Osteoporos Int.

[29] Umberson D, Pudrovska T, Reczek C (2010). Parenthood, childlessness, and well-being: a life course perspective. J Marriage Fam.

[30] Li N (2002). Prevalence rate of osteoporosis in the mid-aged and elderly in selected parts of China. Chin Med J.

[31] Mun-San Kwan M (2011). Falls incidence, risk factors, and consequences in Chinese older people: a systematic review. J Am Geriatr Soc.

